# Metabolic depression and non-specific immune response during hibernation of common Asian toad, *Duttaphrynus melanostictus*

**DOI:** 10.1242/bio.061789

**Published:** 2025-07-07

**Authors:** Debadas Sahoo, Sibakalyani Acharya

**Affiliations:** ^1^ Post-Graduate Department of Zoology, S.C.S. Autonomous College, Puri, Odisha 752001, India; ^2^ Post-Graduate Department of Life Sciences, S.K.C.G. Auto College, Paralakhemundi, Odisha 761200, India

**Keywords:** Hibernation, Metabolic Depression, Non-specific immune response, Leucocytes, Bacteria-killing ability (BKA), Reactive oxygen species (ROS)

## Abstract

To assess metabolic depression and non-specific immune response during hibernation in male common Asian toads, *Duttaphrynus melanostictus,* we measured activities of different enzymes of both aerobic (oxygen-dependent) and anaerobic (oxygen-independent) metabolic pathways in liver tissue and some non-specific immune responses in blood and liver tissue by obtaining hibernating toads directly from their hibernaculum in nature. Though decreased activities of enzymes and suppressions of non-specific immune responses were hypothesised, some contrasting results were found. Activities of citrate synthase (CS) and isocitrate dehydrogenase (ICDH) enzymes of aerobic metabolic pathways showed a significant decrease in their activities during hibernation up to 29% and 61% respectively of their active period value. Contrary to our hypothesis enzymes of oxygen-independent metabolic pathways i.e. pyruvate kinase (PK) and lactate dehydrogenase (LDH) showed no significant changes in their activities during hibernation compared to the active period. This shows aerobic metabolic depression during normoxic hibernation in common Asian toads and maintenance of vital activities at a minimum level with use of energy (ATP) generated from the oxygen-independent metabolic pathway. Likewise, the non-specific immune response comprising total leucocyte count, individual leucocytes like neutrophil, eosinophil, basophil, lymphocyte and monocytes showed a significant decrease in their count during hibernation along with a reduction in complement proteins indicated by serum bacteria-killing ability, compared to active period. In contrast, the levels of reactive oxygen species (ROS) in liver tissue resulting in oxidative stress in terms of TBARS formed and GSSG/GSH ratio were significantly higher during hibernation, suggesting some components of non-specific immunity remain elevated. We conclude that, though there is suppression of non-specific immune response during hibernation to a maximum extent to conserve energy, some components of it in terms of oxidative stress are still in an active state to provide the signal to adaptive immunity for a quick response that is expected during post hibernation phase. Further, it indicates that non-specific immune response during hibernation is variable and tissue-specific.

## Teaser

**Summary:** Metabolic depression and suppression of non-specific immune response was found in hibernating male common Asian toads with elevated oxidative stress markers in liver tissue indicating tissue-specific immune response.

## INTRODUCTION

Alterations in environmental temperature during the cold winter months and a decrease in food availability pose a thermo-regulative challenge for both endothermic and ectothermic animals. Where some homeothermic mammals go heterothermic and drop their body temperature to the surrounding temperature, some amphibians show additional metabolic depression and decrease in body temperature for enduring the winter season by a process called hibernation. Though most ectothermic animals show a decrease in their body temperature with decreased environmental temperature and hypometabolism as per the Q_10_ effect, some of them show additional intrinsic depression in metabolism (Withers and Cooper, 2010). *Ranatemporaria* has been reported to decrease its metabolism by nearly 75% during hibernation in a hypoxic condition (Boutilier and Pierre, 2002). Likewise, Moberley (1963) reported metabolic depression up to 50% of the normal metabolic rate in hibernating desert iguanas (*Dipsosaurus dorsalis*). By suppressing the metabolism and concomitantly decreasing the body temperature, hibernators conserve energy to survive in cold weather with no food availability.

The common Asian toad, *Duttaphrynus melanostictus* (Anura: Bufonidae), native to South Asian countries, hibernates inside burrows (Pratihar and Kundu, 2010; Lin et al., 2011) in moist and loose soil or under leaf litter and debris during winter months (December and January). Like hibernation, estivation is a dormant state shown by many animals to endure dry and hot summers with food and water scarcity (Wilsterman et al., 2020). Both hibernation and estivation are the state of dormancy shown by many animals with reduced metabolic rates and decreased activities to conserve energy for enduring harsh environmental conditions with low energy expenses. Metabolic depression has been reported in estivating burrowing frogs (*Cyclorana* and *Neobatrachus*), indicated by low oxygen consumption and thyroid activity (Seymour, 1973; Withers and Thompson, 2000). Similarly, common Asian toads have been noted for their low oxygen consumption, low body temperature and low thyroid activity during hibernation (Pratihar and Kundu, 2009). Anurans have been reported to undergo metabolic depression during dormancy both in hypoxic (Rossi et al., 2020) and normoxic conditions (Moreira et al., 2020). The common Asian toad that hibernates in a normoxic microhabitat (Patnaik and Sahoo, 2021) is susceptible to aerobic metabolic depression. However, metabolic depression, as evidenced by the activity of some aerobic oxidative metabolic enzymes during the hibernation of common Asian toads, has not been reported. Citrate synthase and Isocitrate dehydrogenase enzymes’ activities, normally considered as the hallmarks of aerobic mitochondrial activity, have not been assessed during hibernation in *Duttaphrynus melanostictus*. Moreover, the activities of enzymes catalysing oxygen-independent ATP synthesis pathways have also not been investigated during hibernation. So the low oxygen consumption during hibernation of the common Asian toad reported by Pratihar and Kundu (2009) has not been substantiated by investigating the changes in activities of some key oxidative metabolic enzymes.

Since hibernation has been well studied in endothermic mammals, there are several reports regarding metabolic depression with decreased body temperature, low oxygen consumption, low activities of metabolic enzymes and alterations in immune responses during hibernation in endothermic mammals (Chazarin et al., 2019; Abnous and Storey, 2008; Storey, 1997, 1987; Stenvinkel et al., 2013; Huber et al., 2021; Sahdo et al., 2013). However, studies concerning immune status during hibernation in amphibians have not been done. In general, different components of the immune system of amphibians are very similar to that of endothermic mammals (Robert and Ohta, 2009) and in both cases, non-specific immune responses provide the first line of defence against the pathogens. Amphibians especially common Asian toads, being hibernators inside moist burrows or under debris, are likely to face pathogenic attacks due to bacteria and fungi. The non-specific immune system consisting of an exterior barrier, phagocyte cells, complement proteins, proteolytic enzymes, and cytokines impart the first line of defence against pathogens. In response to pathogenic attack, macrophages (local inflammatory cells) produce cytokines [interleukins (IL) like IL 1, IL 6 and tumour necrosis factor-alpha (TNF-alpha)], which again induces the liver to secrete some plasma proteins like C-reactive protein (CRP) that binds with phosphorylcholine component of bacterial cell wall for opsonisation and activation of complement pathways to kill the bacteria. Non-specific immunity with serum complement has been reported to be more diverse and functional in ectothermic animals than endothermic vertebrates (Zhu et al., 2005). Likewise, crocodilian serum has been shown to have a higher degree of bacteria-killing ability (BKA) than human serum (Merchant et al., 2003). Similarly, Sunyer et al., (1997) have reported high serum complement protein in teleost fishes. Compared to whole blood serum, bacteria-killing ability (BKA) provides information about complement protein status and their activities indicating innate immune function (Merchant et al., 2003; Terrell et al., 2013). Circulating leucocytes, especially neutrophils and lymphocytes, show significant changes in their proportion in response to stress (Bennette et al., 1972) as well as towards infection (Gervasi et al., 2014), which also indicates their role in the innate immunity of different vertebrates. Likewise, reactive oxygen species (ROS) produced by the NADPH oxidase complex also play a very important role in killing pathogens and occupy the central position in innate immunity. Leto and Geiszt (2006) have reported the crucial role of microbicidal ROS produced by NADPH for host defence. It is well known that after ingesting microorganisms into phagosomes, activated NADPH-oxidase produces NOX-derived superoxide or ROS for phagocytosis (Flannagan et al., 2009). Besides this, ROS play an important role in antigen cross-presentation and have been reported to play an important role in the chemotaxis of leucocytes to the site of infections and signalling function (Hattori et al., 2010). Oxidative stress caused by the overproduction of ROS and weak antioxidant defence has been reported to play a dual role in infections (Lauridsen, 2019). ROS protect the host body by killing the invading microorganisms while also causing tissue damage during inflammation. Oxidative stress results in development and perpetuation of inflammation and thus plays a crucial role in immune response (Lugrin et al., 2014). Thus, the status of oxidative stress acts as an indicator of a non-specific immune response (Lauridsen, 2019; Kozlov et al., 2021).

*Duttaphrynus melanostictus* is a good model species for investigating non-specific immune responses during hibernation due to its easy availability in its natural habitat and available research regarding oxidative stress and antioxidant defence during hibernation (Sahoo and Patnaik, 2020; Patnaik and Sahoo, 2021) and some reports regarding its ageing and physiology (Sahoo and Kar, 2014, 2015, 2017; Nayak et al., 2007). In this study, activities of some key metabolic enzymes like citrate synthase (CS), isocitrate dehydrogenase (ICDH), pyruvate kinase (PK) and lactate dehydrogenase (LDH) were investigated in the liver tissue of hibernating common Asian toads to elucidate their response to low oxygen consumption and low body temperature. Non-specific immune status in terms of blood leucocyte profile, the status of oxidative stress markers and serum bacteria-killing ability (BKA) were also investigated in hibernating toads.

We hypothesised that hibernating common Asian toads might show reduced activities of key metabolic enzymes in response to low oxygen consumption, low body temperature and immobility. To survive with a low energy budget, suppression of non-specific immune parameters was expected. It was also presumed that some components of the non-specific immune response might remain active to protect the animal from pathogens during hibernation.

## RESULTS

### Metabolic enzymes’ activities

**Fig. 1.**
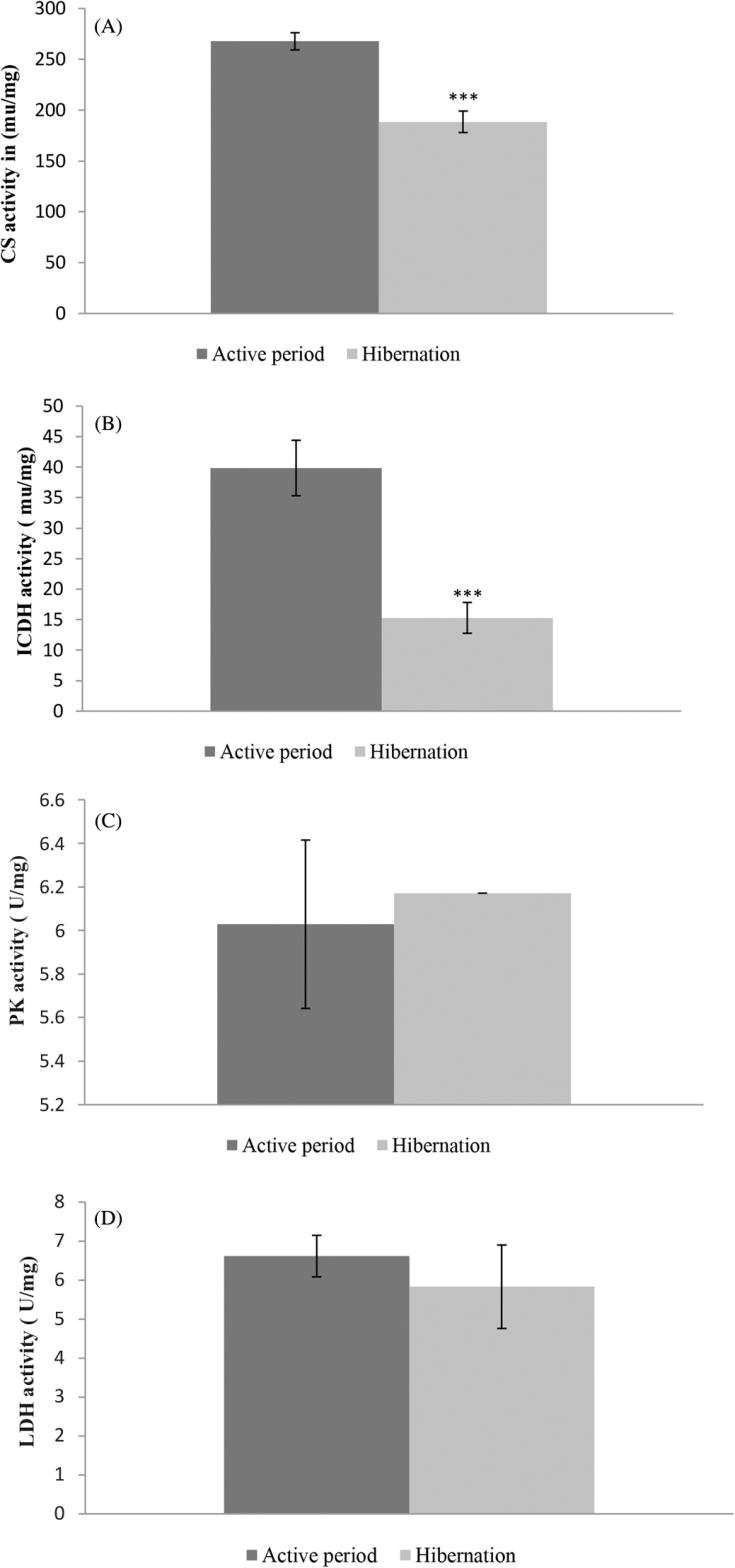
**Activities of key metabolic enzymes in liver tissue of hibernating and active common Asian toads.** (A) Citrate synthase (CS), (B) isocitrate dehydrogenase (ICDH), (C) pyruvate kinase (PK), (D) lactate dehydrogenase (LDH). Animals used in each group (*n*)=7; *** denotes significant differences between hibernating and active toads group at *P*<0.001.

Metabolic enzymes’ activities were found to be lower during the hibernation period than the active period. Citrate synthase (CS), an enzyme in the aerobic metabolic pathway, was found to be at a significantly at a lower level (t=5.84, d.f.=12, P<0.0001) in its activities in the liver tissue of hibernating common Asian toad than in active toads (Fig. 1, Table 2). Likewise, the isocitrate dehydrogenase (ICDH) enzyme also showed significantly (t=4.73, d.f.=12, *P*=0.0005) low activities in liver tissue during the hibernation period in comparison with the active period (Fig. 1, Table 2). A decrease of nearly 29% in citrate synthase (CS) and 61% in isocitrate dehydrogenase (ICDH) activity during the hibernation period compared to the active period was observed in this study. Contrary to enzymes of aerobic metabolic pathways, enzymes of oxygen-independent metabolic pathways were found with almost the same activities during the hibernation period and the active period. Enzymes like pyruvate kinase (PK) (t=0.322, d.f.=12, *P*=0.753) and lactate dehydrogenase (LDH) (t=0.102, d.f.=12, *P*=0.9204) of anaerobic metabolic pathways were found with no significant change in their activities during hibernation compared to the active period. (Fig. 1, Table 2). The protein content of liver tissue was found to have no significant change during the hibernation period compared to the active period.

### Non-specific immune response

**Fig. 2.**
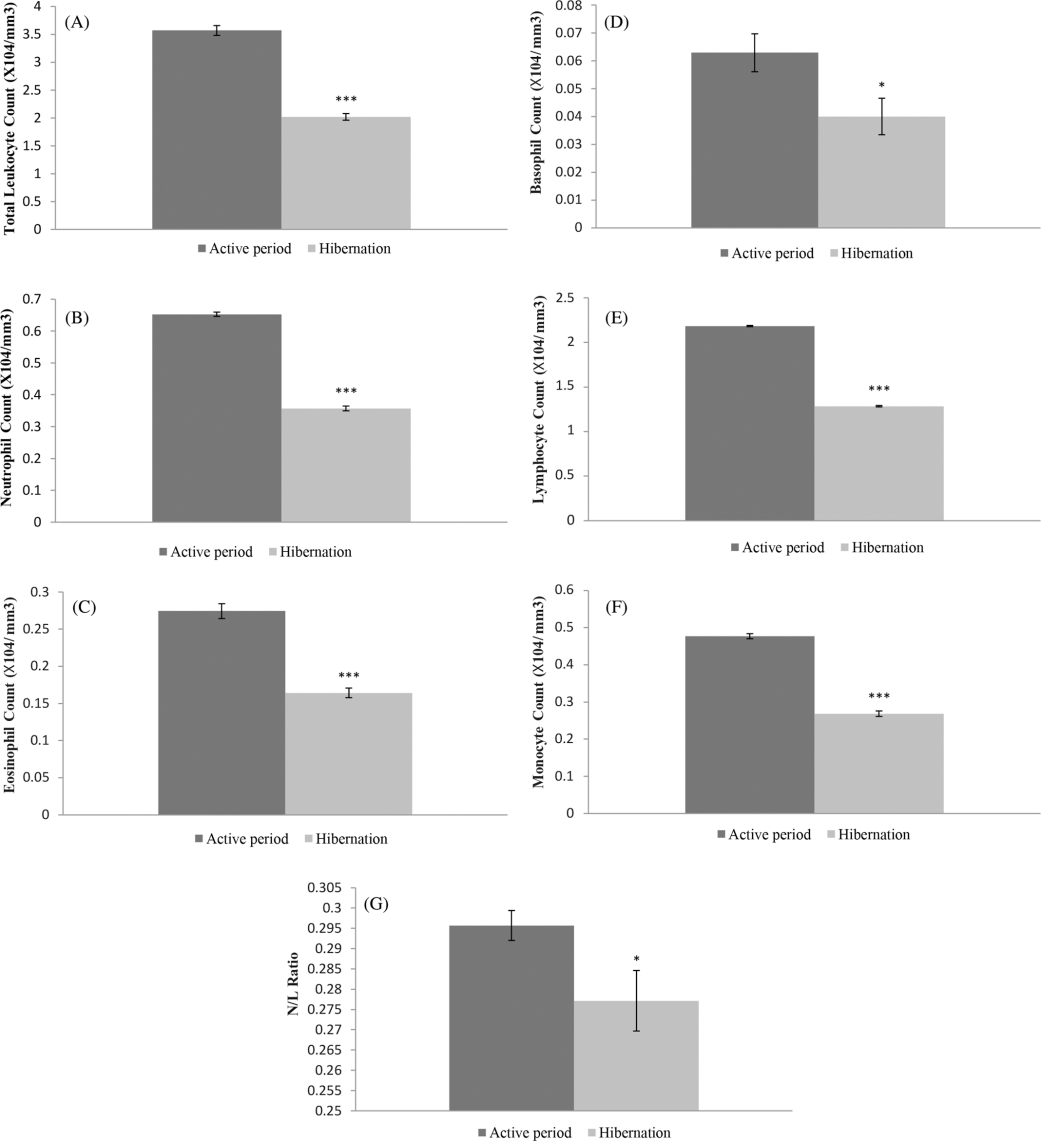
**Non-specific immune status in terms of total leucocyte count, differential leucocyte count and neutrophil/lymphocyte ratio of the blood of hibernating and active common Asian toads.** (A) Total leucocyte count, (B) neutrophil count, (C) eosinophil count, (D) basophil count, (E) lymphocyte count, (F) monocyte count, (G) neutrophil/lymphocyte (N/L) ratio. Animals used in each group (*n*)=7; ***denotes significant differences between hibernating and active toads group at *P*<0.001; *denotes significant differences between hibernating and active toads group at *P*<0.05.

Apart from increased ROS generation, some non-specific immune responses were found to decrease during the hibernation period compared to the active period. Total leucocyte count during hibernation was found to be at a significantly lower level (t=14.55, d.f.=12, *P*<0.0001) than in the active period (Fig. 2, Table 2). It was nearly a 43% decrease on the active period value. Likewise, significantly low levels of neutrophil (t=29.2742, d.f.=12, *P*<0.0001) with 45% decrease, eosinophil (t=9.2474, d.f.=12, *P*<0.0001) with 41% decrease, basophil (t=2.8968, d.f.=12, *P*=0.0134) with 43% decrease, lymphocyte (t=86.6728, d.f.=12, *P*<0.0001) with 41% decrease and monocyte (t=20.7866, d.f.=12, *P*<0.0001) with 43% decrease in their count during the hibernation compared to the active period were found (Fig. 2, Table 2). The neutrophil: lymphocyte (N/L) ratio was also found to be at a significantly lower level (t=2.2295, d.f.=12, P=0.0457) with a decrease of 3.5% in its value during hibernation (Fig. 2, Table 2).

**Fig. 3.**
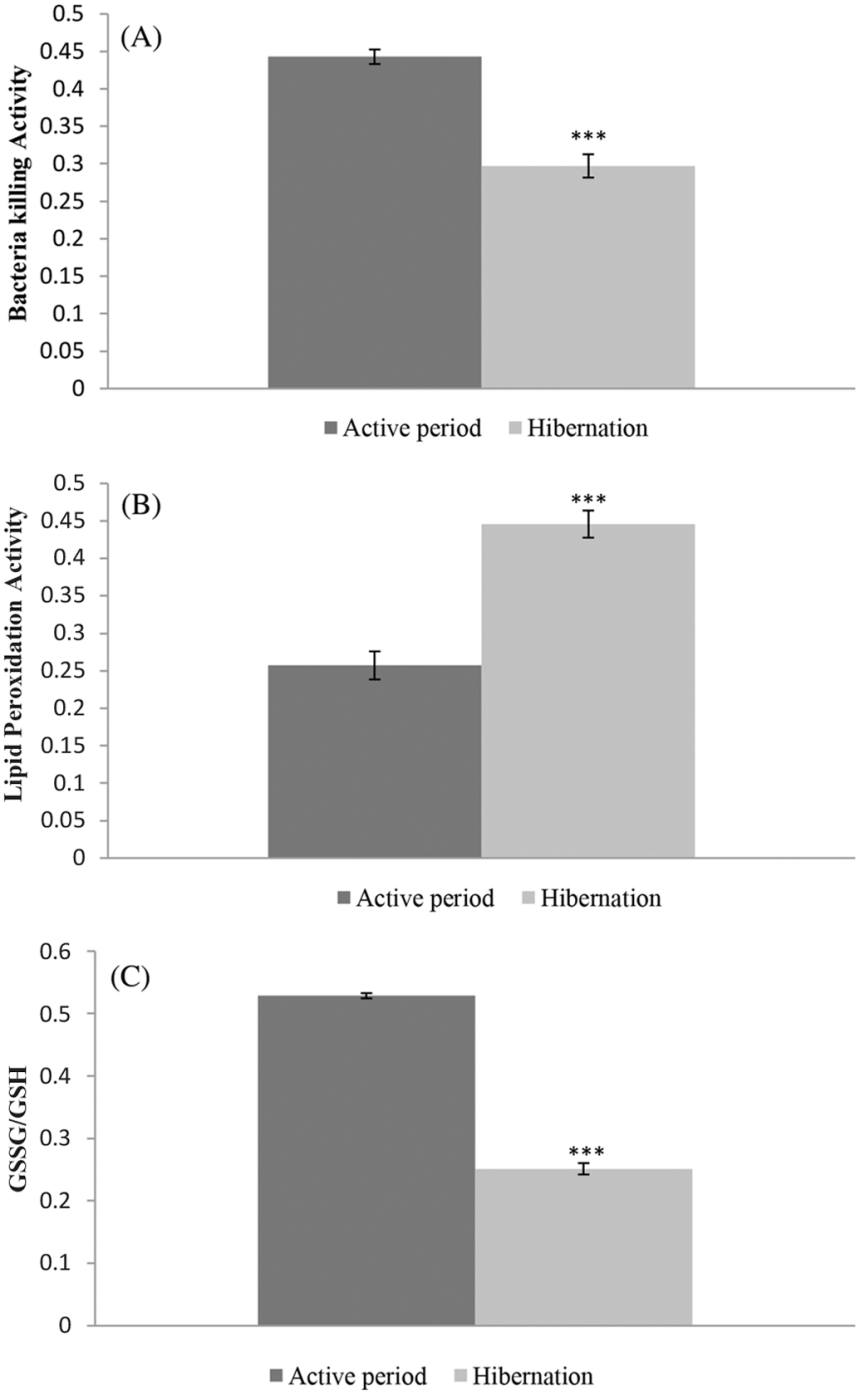
**Non-specific immune status in terms of serum complement protein indicated by bacteria-killing ability (BKA), ROS level in liver tissue in terms of lipid peroxidation (LPO) or TBARS (Thiobarbituric acid reactive substance) formed and oxidised glutathione (GSSG)/reduced glutathione (GSH) ratio.** (A) Bacteria killing ability (BKA), (B) LPO (TBARS), (C) GSSG/GSH ratio. Animals used in each group (*n*)=7. ***denotes significant differences between hibernating and active toads group at *P*<0.001.

Bacteria killing ability (BKA) of blood plasma showed significantly (t=7.95, d.f.=12, *P*<0.0001) lower value during hibernation than the active period with a decrease of 33% (Fig. 3, Table 2).

Unlike leucocytes, the status of oxidative stress markers level in liver tissue indicating ROS level during hibernation was found to increase compared to the active period. Oxidative stress markers level measured in terms of Lipid Peroxidation (Thiobarbituric Acid reactive substance, TBARS formed) was significantly (t=7.23, d.f.=12, *P*<0.0001) higher in liver tissue during hibernation compared to the active period (Fig. 3, Table 2). Another marker of oxidative stress i.e., GSSG/GSH ratio was also significantly higher (t=10.28, d.f.=12, *P*<0.0001) in liver tissue during hibernation than during the active period (Fig. 3 and Table 2).

## DISCUSSION

In this study, we investigated the activities of some key metabolic enzymes and some non-specific immune responses in the liver tissue and blood of hibernating and active common Asian toads. We found a significant decrease in activities of citrate synthase (29%) and isocitrate dehydrogenase (61%) in the liver tissue of hibernating common Asian toads as hypothesized, indicating a low level of activities of aerobic metabolic enzymes, which have been reported as the proxies for reduced aerobic capacity and mitochondrial activity (Moreira et al., 2020, 2021). Contrary to our hypotheses, pyruvate kinase (PK) and lactate dehydrogenase (LDH) enzymes were found to undergo no significant changes in their activities during hibernation. These enzymes of oxygen-independent metabolic pathways remaining unaltered in their activities indicate steady maintenance of oxygen-independent ATP production capacity. Low citrate synthase (CS) and isocitrate dehydrogenase (ICDH) activities indicating reduced aerobic capacity and mitochondrial activity during normoxic hibernation (Patnaik and Sahoo, 2021) in common Asian toads are in good agreement with the reports by Mantle et al., (2010) and Moreira et al., (2020) in different estivating anurans. Decreased aerobic capacity and mitochondrial activity corroborate with decreased thyroid activity and oxygen consumption in common Asian toads during hibernation (Pratihar and Kundu, 2009) with metabolic depression (Withers and Cooper, 2010). Metabolic depression during the estivation of *Pleurodema diplolister* has also been reported (Madelaire et al., 2020) with increased expression of AMPK to suppress metabolic activity. Likewise, St-Pierre and Boutilier, (2001) have also observed decreased aerobic capacity characterised by suppression of key metabolic enzymes like citrate synthase and cytochrome c oxidase during hibernation of *Rana temporaria*.

Along with reduced aerobic capacity, steady maintenance of oxygen-independent ATP production capacity indicates metabolic depression and continuation of vital life processes with a low energy budget. Unlike hibernation in *Rana temporaria* in hypoxic conditions (St-Pierre and Boutilier, 2001), maintenance of pyruvate kinase (PK) and lactate dehydrogenase (LDH) activities at a steady level during hibernation in the common Asian toad may be due to hibernation in normoxic conditions and production of ATP at substrate level oxidation to manage different vital activity at a very low rate. Decreased activity of enzymes like LDH and pyruvate dehydrogenase has been reported during hibernation of anurans (St-Pierre and Boutilier, 2001) and aestivation of snails (Brooks and Storey, 1992) respectively in a hypoxic or anoxic conditions. Decreased activities of these enzymes have been reported to be due to phosphorylation during hibernation (Brooks and Storey, 1992). Our observation of steady maintenance of PK and LDH activities during hibernation supports previous findings that it results in rapid resumption of normal activity at the end of hibernation with the increase in ambient temperature.

As we hypothesised regarding activities of key enzymes of hibernation metabolism, we found low-level activities of citrate synthase (CS) and isocitrate dehydrogenase (ICDH) (enzymes of oxygen-dependant metabolic pathways) during hibernation with low oxygen consumption and low body temperature. However, contrary to our hypothesis, enzymes of oxygen-independent pathways were found not to undergo any significant changes in their activities. This may be a stop-gap arrangement for the slow production of ATP for continuing important vital activities with a low energy budget. In good agreement with our findings, activities of PK and hexokinase (enzymes of glycolytic pathways) have been reported to be maintained at a steady level during the estivation of African lungfish (Frick et al., 2008). Likewise, LDH activity in the skeletal muscle of estivating *Protopterus dolloi* has also been reported to undergo no significant change (Frick et al., 2008).

Concomitant with metabolic depression, we found decreased non-specific immune response during hibernation in common Asian toads. The total leucocyte count during hibernation was found to be significantly lower than during the active period. Individually different leucocytes like neutrophils, eosinophils, basophils, lymphocytes and monocytes were also found to decrease during hibernation compared to the active period. It may be due to their decreased formation owing to energy conservation during metabolic depression. The decreased heartbeat rate that we observed might be accompanied by reduced blood flow, resulting storage of available leucocytes in lymphoid organs. A significant decrease in the proliferation of T-lymphocytes and mean percentage of eosinophils in *Rana pipiens*, which has been reported earlier (Maniero and Carey, 1997) under low temperature (5°C) exposure for 3 to 5 months corroborates our findings. Cooper et al., (1992), have also reported progressive loss of Lymphomyeloid organs and blood leucocyte populations of Leopard frogs during hibernation at 4°C and their restoration during post hibernation period. Likewise, Greenspan et al., (2017) have reported that the susceptibility of cold-treated (16°C) frog (*Litoriapaerulea*) to the fungal infection by *Batrachochytrium dendrobatidis* was due to a decline in WBC count especially lymphocytes, monocytes, eosinophils and basophils. Rollins-Smith and Wood Hams, (2012) have suggested increased immune function at warm temperatures and its decrease at low temperatures with metabolic depression and a shorter supply of energy in ectothermic animals. With this line of thought Raffel et al., (2015) have reported a higher rate of fungal (*B. dendrobatidis*) infection in Cuban tree frogs and red-spotted newts when they were shifted from 25°C to 15°C compared to the shifting from 15°C to 25°C. Our findings regarding decreased total leucocyte count during hibernation with low body temperature and metabolic depression are in good agreement with the findings of Wright and Cooper, (1981), Zapata et al., (1992), Rollins Smith and Wood Hams, (2012). Hibernation being the state of dormancy with reduced metabolism has been reported with severe depletion of circulating lymphocytes in mammalian hibernators, Syrian hamsters (Bouma et al., 2011). They have also reported storage of lymphocytes in secondary lymphoid organs owing to temperature-dependent decrease in plasma sphingosine–1–phosphate (S1P) level.

Since there was a significant decrease in total leucocyte count, we found the ratio of neutrophil to lymphocyte (N/L) significantly decreasing during hibernation in common Asian toads compared to their counterparts during the active period. It has been suggested that the leucocyte profile is the parameter for hematopoietic productivity and level of immune system activation (Davis et al., 2008). The ratio between neutrophil to lymphocyte (N/L) has also been suggested as a reliable indicator of the status of the immune system (Davis et al., 2008). The decreased N/L ratio that we found in this investigation indicates a suppressed immune response during hibernation. The low N/L ratio that has been reported to be an indicator of decreased immunity in *Litoriacaerulea* (Greenspan et al., 2017) corroborates our finding.

In ectothermic animals, serum complement proteins have been reported to be more functional (Zhu et al., 2005). Serum complement protein status in terms of serum bacterial killing ability (BKA) was determined in this study in both hibernating and active toads. Compared to active toads, hibernating toads had low bacteria-killing ability in their serum, indicating a low level of complement proteins. This may be due to their low rate of synthesis in a hypometabolic condition. The low rate of BKA activity of blood plasma at a lower temperature gradient (5-10°C) than a higher temperature gradient (10-18°C) from bullfrogs kept at 28°C for 83 days (Lima et al., 2020) corroborates our finding. Likewise, the decrease in T-lymphocyte proliferative ability (Green and Cohen, 1977), complement activity (Maniero and Carey, 1997) and inhibition of anti-microbial peptide synthesis (Matutte et al., 2000) has been reported in hibernating and cold-exposed frogs like *Rana pipiens* and cold-treated (5°C) *Rana sylvatica*.

Unlike leucocyte and BKA, reactive oxygen species (ROS) levels were found to be increased in the liver tissue of hibernating common Asian toads compared with active toads. Both TBARS content and GSSG:GSH ratio were comparatively higher in the liver tissue of hibernating toads than in active toads, indicating a higher level of ROS during hibernation. Our previous study (Patnaik and Sahoo, 2021) regarding oxidative stress markers and anti-oxidant defence reported increased ROS levels in both liver and brain tissue during hibernation, which is in good agreement with Grundy and Storey, (1998); Carey et al., (2000); Avci et al., (2014); Prokić et al., (2019) and Niu et al., (2022). Reduced oxygen consumption and metabolic depression resulting in the reduced redox state of the mitochondrial electron transport system and generation of superoxide radicals (ROS) have been reported (Hernansanz-Agustín et al., 2014) to be among the causes of the production of ROS during low oxygen consumption. Increased levels of ROS during hibernation act as a non-specific immune response and provide a signal to the adaptive immune system to respond. Though immune suppression in terms of low leucocyte count and low bacteria-killing ability were found in the blood during hibernation, increased ROS levels in liver tissue indicates its importance for maintaining non-specific immunity at elevated levels. The importance of ROS concerning immune system processes like proliferation, differentiation, intracellular signalling, chemoattraction and antigen cross-presentation has been reported (Tavassolifar et al., 2020). Immune suppression in terms of decreased leucocyte count and low bacteria-killing ability that were observed in blood and elevated ROS levels in the liver during hibernation indicates differences in immune status in different tissues. When most of the components of the non-specific immune system are in suppressed condition, at least some components are at elevated levels to counteract the expected infections. This is consistent with our hypothesis. Moreover, elevated ROS in liver tissue during hibernation is capable of providing the signal to adaptive immunity to show a quick response that is supposed to be needed during the post-hibernation phase with susceptibility towards infection. When the leucocyte profile shows immune suppression during hibernation, how the liver maintains an increased level of ROS requires further investigation. However, from this investigation, it is suggested that the immune status especially non-specific immunity does not remain static in different organs rather it remains at different levels in different organs during hibernation.

### Conclusion

Hibernation in common Asian toads, an adaptive response towards decreased ambient temperature and scarcity of food material, was observed to have intrinsic metabolic depression characterised by the suppression of aerobic (oxygen-dependant) metabolic enzymes’ activity. Enzymes of the oxygen-independent pathway were found with no change in their activity indicating energy (ATP) production using a glycolytic pathway, though in a lower amount. Although in a depressed metabolic condition during hibernation, non-specific immunity comprising leucocyte status and bacteria-killing ability of blood were in suppressed condition, the status of ROS level in liver tissue was in the elevated state compared to the active period. This shows that immune response during a hypometabolic condition like hibernation is tissue specific.

## MATERIALS AND METHODS

### Animal collection and experimental conditions

Animal collection and treatments were as per the instructions of the institutional animal ethics aommittee, Berhampur University, India with registration number 2020/Go/Re/S/18/CPCSEA vide resolution number 01.

Male common Asian toads, *Duttaphrynus melanostictus* of about 4 years old (snout–vent length of 8.0-8.4 cm and body weight of 37-52 gm) were collected from their natural habitat (a protected area with boundary wall surrounding abandoned houses, uncared-for gardens, swampy area with mouse holes and old rotten logs and bamboo) located in Paralakhemundi (10^0^ 45′ N. 84^0^ 6′ E), India, for this study. The determination of age and sex was based on our previous report (Sahoo and Kara, 2017; Patanaik and Sahoo, 2021). Briefly, the age of the individuals was ascertained by skeletochronology i.e. counting the lines of arrested growth (LAG) in the long bone matrix (Smirina, 1972), which has a positive correlation with snout to vent length of the body (Sahoo and Kara, 2017). Males were identified by observing a brick red or orange-coloured hue on the throat region and black nuptial pads on the two inner fingers of the forelimb. Considering the egg-laying capacity of females to increase the population, they were not chosen for this study. Morphometric parameters of toads collected during different periods like the active summer period and hibernation period for comparison of different key enzymes’ activities of metabolism and nonspecific immune parameters were given in Table 1. In this study, summer active toads (*n*=7) were collected during the night hours (nocturnal animal) of June to August 2021 (summer with rainfall) and Hibernating toads (*n*=7) were collected during the first week of January 2022 (mid-winter) by observing their cement-grey-coloured immobile body with dried mucus from their burrows (hibernaculum) of 30-70 cm deep during night hours for comparison of some non-specific immune status. They had been under observation since early winter in their foraging ground. With a gradual decrease in environmental temperature, they started disappearing from the ground. After a lot of effort, we found them under wooden logs or inside burrows (hibernaculum) even during night hours indicating the starting of hibernation. For comparison of activities of some key metabolic enzymes (CS, ICDH, PK and LDH) in liver tissue of active toads and hibernating toads, they were collected from their natural habitat/hibernaculum during August to September 2024 (active period) and the last week of December 2024 (hibernation period) respectively.

**Table 1.** Morphometric parameters of *Duttaphrynus melanostictus,* collected during the summer active period and hibernation period

Condition of the Toad	Period of collection	Atmospheric temperature	Humidity %	Snout to vent length (Cm)	Body weight (g)	Body temperature	Heart beat rate (times/min)
Summer Active	June to August 2021	36°C±4°C	72±5	8.0-8.2	37-52	27±1°C	41–48
	August to September 2024	35°C±4°C	76±4	8.0-8.2	37-52	27±1°C	41–48
Hibernation	First week of January 2022	9°C±3°C	34±4	8.0-8.2	31-45	9±1°C	11–14
	Last week of December 2024	8°C±3°C	34±4	8.0-8.2	31-45	9±1°C	11–14

Data regarding atmospheric temperature and humidity are from the District Administration, Gajapati, Odisha, India.

**Table 2.** Variations in activities of key metabolic enzymes and non-specific immune parameters during the active period and hibernation period in common Asian toad

Tissue	Parameters	Active period	Hibernation period	Percentage (%) of change
Liver	Citrate synthase (CS) activity	267.71±8.47	188.57±10.55***	–29%
	Isocitrate dehydrogenase (ICDH) activity	39.86±4.54	15.29±2.52***	–61%
	Pyruvate kinase (PK) Activity	6.03±0.39	6.24±0.53	+4%
	Lactate dehydrogenase (LDH) activity	6.61±0.53	6.69±0.52	+1%
	Lipid peroxidation (LPO)	0.26±0.02	0.446±0.018***	+72%
	GSSG/GSH ratio	0.15±0.004	0.25±0.009***	+67%
Blood	Total leucocyte count (×10^4^/mm^3^)	3.57±0.09	2. 02±0.06***	–43%
	Neutrophil count	0.65±0.007	0.36±0.008***	–45%
	Eosinophil count	0.27±0.01	0.16±0.007***	–41%
	Basophil count	0.07±0.007	0.04±0.009*	–43%
	Lymphocyte count	2.18±0.007	1.28±0.008***	–41%
	N/L tatio	0.29±0.004	0. 28±0.007*	–3.5%
	Monocyte count	0.47±0.006	0.27±0.007***	–43%
	Bacteria-killing ability (BKA)	0.443±0.009	0.04±0.009***	–33%

Data are expressed as mean±s.e.m. animals used in each group (*n*)=7; * indicates a significant difference at *P*<0.05; *** indicates a significant difference at *P*<0.001.

### Tissue preparation

Both summer active and hibernating (*n*=7) common Asian toads were collected from their natural habitat during night hours and immediately decapitated to dissect and take out the whole liver and stored in ice-cold (2°C) Amphibian Ringer's solution, which was prepared in the laboratory by mixing NaCl, KCl, CaCl_2_, NaHCO_3_ in distilled water. They were then transferred immediately to the laboratory (700 m from the collection site) for further processing. Adherent tissues were removed from the liver, weighed and processed for estimation of enzyme activities and oxidative stress markers. Likewise, blood was collected from the aorta using a 1 ml syringe with a 26 g needle for investigating non-specific immune parameters.

### Measurement of enzymatic activities

For assay of enzyme activities, 2.5% (w/v) tissue homogenate in ice-cold 50 mM Triss buffer (pH=7.4) (HiMedia, GRM1218), 1 mM EDTA (HiMedia, MB011) and 1 mM phenyl methyl sulphonyl fluoride (HiMedia and SRL, MB144) prepared inside precooled Teflon-glass tissue homogenizer was centrifuged at 10,000×***g*** for 15 min at 4°C. Immediately the supernatant was kept in the ice bucket and used for enzyme activities assay. Enzyme activities assay was performed for both active toads and hibernating toads at 25°C using a UV – Vis spectrophotometer (Systronics -119).

#### Citrate synthase (CS)

Measurement of CS activity was done following Srere, (1969) by measuring the formation of 5 – thio – 2 nitrobenzoic acid (TNB) at 412 nm from the reaction between DTNB (5,5′, Dithio – bis – 2 – nitrobenzoic acid) and SH group of co-enzyme A. Briefly 50 mM triss buffer (pH 8.0), 0.1 mM acetyl-Co A, 0.5 mM Oxaloacetate, 0.1 mM DTNB and 1:1500 (v/v) tissue sample were in the experimental condition and one of the control was without tissue sample and another was without oxaloacetate. CS activity was measured by recording the decrease in OD for 2 min and expressed in unit/mg protein where one unit causes the production of one **µ** mol. of TNB/minute with a molar extinction coefficient of 14.15 mM^−1^ cm^−1^.

#### Isocitrate dehydrogenase (ICDH)

ICDH activity was measured by estimating the formation of NADPH at 340 nm following Fox (1971). Briefly 50 mM imidazole buffer (pH=7.2), 5 mM MgSO_4_, 0.4 mM NADP^+^, 2 mM isocitrate and 1: 200 (v/v) tissue samples were in the experimental condition and one of the control assays was without tissue sample and another one was without isocitrate. Enzyme activity was measured by recording the OD for 1-5 min and expressed as units/mg protein where one unit of ICDH produces 1 µmol of NADPH/min measured as per molar extinction coefficient of 6.22 mM^−1^ cm^−1^.

#### Pyruvate kinase (PK)

PK activity was measured following methods as described by Weber (1969) i.e. by estimating consumption of NADH at 340 nm. In the experimental condition 50 mM imidazole buffer (pH=7.2), 1 mM ADP, 100 mM KCl, 0.2 mM NADH, 5 mM MgSO4, 1 mM Phosphoenolpyruvate, 3 U/ml Lactate dehydrogenase and 1: 40,000 (v/v) tissue sample were taken whereas one control was without tissue sample and another was without phosphoenolpyruvate. Enzyme activity was measured by recording the OD for 3 min and expressed as units per mg protein where 1 unit of PK represents the amount that consumes 1 µmol NADH/min measured using the molar extinction coefficient of 6.22 mM^−1^ cm^−1^.

#### Lactate dehydrogenase (LDH)

LDH activity was measured by following the method as described by Cowan et al., (2000) i.e. by estimating the consumption of NADH at 340 nm. Briefly in the experimental condition, 50 mM imidazole buffer (pH=7.2), 0.2 mM NADH, 2 mM Pyruvate, 5 mM Dithiothreitol and 1: 40,000 (v/v) tissue samples were taken and one of the control was without tissue sample and another one was without pyruvate. Enzyme activity was measured by recording the decrease in OD for 5 min and expressed in units/mg protein where 1 unit of LDH activity represents 1 µmol NADH consumed/minute by the enzyme, measured by using the molar extinction coefficient of 6.22 mM^−1^ cm^−1^.

The protein content of the supernatant obtained by centrifugation of tissue homogenate was estimated following Lowry et al., (1951) using Bovine serum albumin as standard.

### Non-specific immunoassay

#### Blood collection and leucocyte count

Soon after the animal was dissected, the blood was collected from the aorta into a k2-EDTA anticoagulant blood collection tube using a 1 ml syringe with a 26 g needle. A small drop of blood (5 µl) was taken on a glass microscopic slide to make a thin uniform smear for differential leucocyte count. In a WBC pipette, blood was sucked up to 0.5 mark and then WBC diluting fluid was sucked up to 11 mark (one part blood: 20 parts diluting fluid), thoroughly mixed and a drop of diluted blood was taken on the haemocytometer slide for total leucocyte count. The remaining blood was stored in an ice bucket and centrifuged at 2000×***g*** for 5 min to isolate plasma for bacteria-killing assay. The blood smear was air-dried, stained with methanolic Giemsa's stain and observed under the light microscope. For each smear neutrophils (N), eosinophils (E), basophils (B), lymphocytes (L), and monocytes (M) were identified (Heatley and Johnson, 2009) and their number was counted out of 100 leucocytes and recorded as percentages of total leucocytes. The absolute number of each type of leucocyte was calculated from the total number of leucocytes using the percentage count of individual leucocytes (Blumenreich, 1990). The ratio of neutrophils to lymphocytes (N/L) number was also calculated to assess the status of non-specific immune response.

#### Bacteria killing assay

Bacteria killing ability of blood plasma was done against *E. coli* (ATCC8739) following Terrell et al., (2013) modified from Liebl and Martin, (2009). On a 5% blood agar plate (defibrinated sheep blood, ThermoFisher Scientific, catalogue code R54012) a colony of *E. coli* was isolated and used to inoculate a stock solution. By plating serial dilutions (10^−4^, 10^−5^, 10^−6^) onto 5% blood agar, the bacterial concentration of the stock solution was calculated. For each sample 5 µl of fresh blood plasma, 20 µl of bacteria (diluted to 10^6^ colony forming Unit/ml) and 75 µl of amphibian phosphate buffered saline were combined in duplicate in a 96-well plate. Plates were placed in a Shaker incubator (1 h at 21°C) for incubation and bacteria killing. Tryptic soy broth (200 µl) was added to each well and mixed manually by pipetting. Absorbance was read at 405 nm after a period of incubation of plates at 30°C for 12 h for bacterial growth using a plasma blank (plasma and phosphate buffer saline), a negative control (Tryptic soy broth and phosphate buffer saline) and a positive control without plasma. Bacteria killing ability was determined as follows: BKA=A sample–A plasma blank; A positive control, where A is the absorbance at 405 nm.

#### Measurement of oxidative stress markers

Oxidative stress markers are the molecules that are modified by the action of reactive oxygen species (ROS) and also the antioxidant molecules that change in response to increased redox stress. Since, direct measurement of ROS levels with high accuracy is difficult due to their high reactivity and short life span, indirect measurement of ROS levels by assessing the oxidative stress they cause is an encouraging alternative (Katerji et al., 2019). Assessment of oxidative stress markers like lipid peroxidation, protein carbonylation, hydroxylation of deoxyguanosine residue of DNA, and antioxidant couple (GSSG/GSH) have been reported as the alternative way to find out ROS level (Bagnyukova et al., 2002; Katerji et al., 2019). Oxidative stress evidenced with raised GSSG/GSH ratio has been reported in many cellular systems (Nemeth and Boda, 1989; Wu and Yetnda, 2011; Sentellas et al., 2014) and also in hibernating frogs and toads (Niu et al., 2018; Patnaik and Sahoo, 2021; Niu et al., 2022). In this study lipid peroxidation assay and GSSG/GSH assay were undertaken to estimate oxidative stress markers level in liver tissue of hibernating common Asian toad.

#### Lipid peroxidation

Lipid peroxidation in terms of TBARS (Thio Barbituric Acid reactive substance) formed was measured following Sestini et al. (1991). 0.5 ml of 2.5(w/v) tissue homogenate with 50 mM Potassium Phosphate Buffer (pH 7.0) and 0.5 mM EDTA and a few crystals of phenyl methyl sulfonyl fluoride, 0.5 ml of 0.6% TBA (Sigma-Aldrich, USA, catalogue code MB144) and 1.5 ml of 1% orthophosphoric acid were heated in an experimental test tube at 95°C for 45 min whereas control tube was with distilled water instead of tissue homogenate. Both the sets were cooled to room temperature, added with 3 ml of chloroform and 1 ml of glacial acetic acid and centrifuged at 1000×***g*** for 10 min. Extinction of the upper phase of the supernatant (TBARS) was measured against the control at 535 nm lipid peroxidation level was expressed as µmol of TBARS formed/g tissue wet weight using the molar extinction coefficient of 1.56×10^5^ M^−1^ cm^−1^.

### GSSG:GSH ratio

Glutathione consisting of both reduced glutathione (GSH) and oxidised glutathione (GSSG) were measured following Griffith (1980) as described in Patnaik and Sahoo (2021).

### Statistical analysis

Data homogeneity was tested using the Shapiro–Wilk test using IBM, SPSS-25.0 software. Besides that, the student's *t*-test was used to compare the mean of two different groups (active period and hibernation period). Differences were considered significant at *P*<0.05.
